# Supplementary material for: Predicting and Testing Bioavailability of Magnesium Supplements
Source: Nutrients. 2019 Jul 20;11(7):1663. doi: 10.3390/nu11071663 (PMC6683096; doi:10.3390/nu11071663)
Supplement: Supplementary file 1 [file nutrients-11-01663-s001.pdf]

Table S1. qualitative composition of the 15 magnesium formulations used in this study.

Product	Qualitative Composition
Ultractive Magnesium	Magnesium glycerophosphate, magnesium oxide, hydroxypropyl methylcellulose, microcrystalline cellulose, polyvinylpyrrolidone, citric acid, sodium hydrogencarbonate, magnesium stearate, pyridoxine hydrochloride
MagnéVie B6	Anhydrous magnesium citrate, pyridoxine hydrochloride, lactose anhydrous, macrogol 6000, magnesium stearate, hypromellose, titanium dioxide (E171), talc
Promagnor	Magnesium oxide, microcrystalline cellulose, hydroxypropyl methylcellulose magnesium stearate, titanium dioxide
Mag2	Magnesium carbonate, pregelatinised corn starch, potato starch, magnesium stearate, talc
Magnesium Verla N tablets *	Calcium carbonate, dimeticon (350 cSt), glucose syrup, glycerol, potassium, macrogol 35000, macrogol 6000, methyl acrylic acid-ethyl acrylic copolymer D, montan wax, povidone K25, sucrose, talc, titanium dioxide, triethylcitrate, vanillin
Magnerot	Magnesium orotate dihydrate, lactose monohydrate
Mag2 24h	Magnesium oxide, cellulose, calcium phosphate, hydroxypropyl methylcellulose, calcium phosphate, mono and diglycerides of fatty acids, cocoa bean extract, magnesium salts of fatty acids, silicon dioxide, Vitamin B6, sodium carboxymethylcellulose
Polase	Microcrystalline cellulose, potassium citrate, magnesium citrate, croscarmellose sodium, glycerol behenate, polyvinylpyrrolidone, magnesium stearate, silicon dioxide
Biolectra	Magnesium oxide, gelatine, starch pregelatinised, polyethylene glycol, titanium dioxide
High Absorption Magnesium	Magnesium glycinate lysinate chelate, microcrystalline cellulose, croscarmellose sodium, magnesium stearate, stearic acid, hypromellose, silicon dioxide, starch, glycerin, hydroxypropyl cellulose
MagOx 400	Magensium oxide, microcrystalline cellulose, hydroxypropyl cellulose, crospovidone, hypromellose, maltodextrin, magnesium stearate, silicon dioxide, PEG
Magnesium 500mg	Magnesium oxide, cellulose, dicalcium phosphate, citric acid, titanium dioxide, magnesium stearate
Magnesium Citrate 200mg	Magnesium citrate, hydroxypropyl cellulose, microcrystalline cellulose, vegetarian coating, croscarmellose sodium, stearic acid, magnesium stearate
Slow Mag	Magnesium chloride hexahydrate, calcium carbonate, cellulose acetate phthalate, pregelatinized starch, povidone, diethyl phthalate, talc, titanium dioxide, magnesium stearate, carnauba wax
B-Magnum	Magnesium oxide, starch, cellulose, calcium phosphate, hydrogenated cotton oil, talc, shellac, hydroxypropyl methylcellulose, titanium dioxide
